# Knockdown of linc-POU3F3 suppresses the proliferation, apoptosis, and migration resistance of colorectal cancer

**DOI:** 10.18632/oncotarget.5830

**Published:** 2015-10-15

**Authors:** Ti-Dong Shan, Ji-Hao Xu, Tao Yu, Jie-Yao Li, Lin-Na Zhao, Hui Ouyang, Su Luo, Xi-Ji Lu, Can-Ze Huang, Qiu-Shen Lan, Wa Zhong, Qi-Kui Chen

**Affiliations:** ^1^ Department of Gastroenterology and Guangdong Provincial Key Laboratory of Malignant Tumor Epigenetics and Gene Regulation, Sun Yat-Sen Memorial Hospital, Sun Yat-Sen University, Guangzhou, Guangdong 510120, People's Republic of China; ^2^ Department of General Surgery, Sun Yat-Sen Memorial Hospital, Sun Yat-Sen University, Guangzhou, Guangdong 510120, People's Republic of China; ^3^ Department of Gastroenterology, the First Affiliated Hospital, Guangzhou University of Traditional Chinese Medicine, Guangzhou, Guangdong 510504, People's Republic of China

**Keywords:** linc-POU3F3, colorectal cancer, proliferation, apoptosis, signal pathway

## Abstract

Long intergenic noncoding RNAs (lincRNAs) play important roles in regulating the biological functions and underlying molecular mechanisms of colorectal cancer (CRC). Here, we investigated the association of linc-POU3F3 and prognosis in CRC. We demonstrated that linc-POU3F3 was overexpressed in CRC tissues and positively correlated with tumor grade and N stage. Inhibition of linc-POU3F3 resulted in inhibition of cell proliferation and G1 cell cycle arrest, which was mediated by cyclin D1, CDK4, p18, Rb, and phosphorylated Rb. Inhibition of linc-POU3F3 induced apoptosis, and suppressed migration and invasion in LOVO and SW480 cell lines. This inhibition also increased the expressions of epithelial markers and decreased the expressions of mesenchymal markers, thus inhibiting the cancer epithelial-mesenchymal transition. The decreased migration and invasion following linc-POU3F3 knockdown were mediated by an increased BMP signal. Furthermore, autophagy was enhanced by linc-POU3F3 knockdown, suggesting the involvement of autophagy in the induced apoptosis. Collectively, linc-POU3F3 might be crucial in pro-proliferation, anti-apoptosis, and metastasis in LOVO and SW480 cells by regulating the cell cycle, intrinsic apoptosis, BMP signaling and autophagy. Thus, linc-POU3F3 is a potential therapeutic target and novel molecular biomarker for CRC.

## INTRODUCTION

Colorectal cancer (CRC) is the fourth leading cause of cancer related death in the world, and the third most frequent cause of cancer-related death in western societies [1, 2]. The incidence and mortality of CRC in some developing countries, such as China, have continued to increase along with their transition towards the so-called western lifestyle, such as the consumption of high-fat diets and physical inactivity. Although efforts have been made to prevent CRC, the incidence of CRC has been increasing for decades [3]. Determining the pathogenic mechanisms and identifying more accurate prognostic biomarkers would not only help CRC prognosis estimations, but also would provide novel potential targets for therapy.

Recently, growing evidence has suggested that epigenetic alterations participate in carcinogenesis and progression of malignancies [4, 5]. Long noncoding RNAs (lncRNAs) generally comprise ribonucleic acid molecules longer than 200 nucleotides without defined open reading frames, which regulate gene expression at epigenetic transcriptional and post-transcriptional levels [6]. Long intervening noncoding RNAs (lincRNAs), a subtype of lncRNAs, are transcript units located within genomic intervals between two protein coding genes [7, 8]. Studies have indicated that abnormal expression of lincRNA occurs in a disease-, tissue-, or developmental stage-specific manner [9, 10]. LincRNAs, as tumor suppressors or promoters, can attenuate or enhance cell proliferation, differentiation, apoptosis, the immune response, and migration in the pathological processes of cancer [11, 12]. Nevertheless, there are thousands of functional lincRNAs yet to be identified.

Recently, efforts have been made to identify lincRNAs systematically in cancer and to explore their functions in tumorigenesis. Aberrant expressions of certain lincRNAs closely correlate with the progression and prognosis of CRC, such as CCAT1-L and HOTAIR [13, 14]. Many signal pathways have been studied to determine the mechanism underlying the proliferation, invasion and metastasis of CRC cells. One important pathway is bone morphogenetic protein (BMP) signaling (including BMPRs, SMAD4, and pSMAD1, 5, 8), which is involved in cellular proliferation, adhesion, differentiation, inflammation, apoptosis, and metastasis in CRC [15]. Autophagy is critical in the defense system against diverse stress conditions, including oxidative stress, nutrient deprivation, growth factor depletion and hypoxia [16–18]. Expression of the autophagy related genes (Atg5, Atg7, Beclin 1, and LC3) generally correlated with the autophagic activity [19, 20].

In the present study, we identified a lincRNA as a novel biological marker in CRC, termed as linc-POU3F3, whose altered expression was previously documented in esophageal squamous cell carcinoma cells (ESCC) and glioma [21, 22]. However, the role of linc-POU3F3 expressions was unexplored in CRC. The objective of our study was to determine the linc-POU3F3 expression patterns between CRC and normal colorectal tissues, and to reveal the function of linc-POU3F3 and the signal pathways involved in CRC cancer cell lines.

## RESULTS

### Increased expression of linc-POU3F3 in human CRC tissues

**Figure 1:**
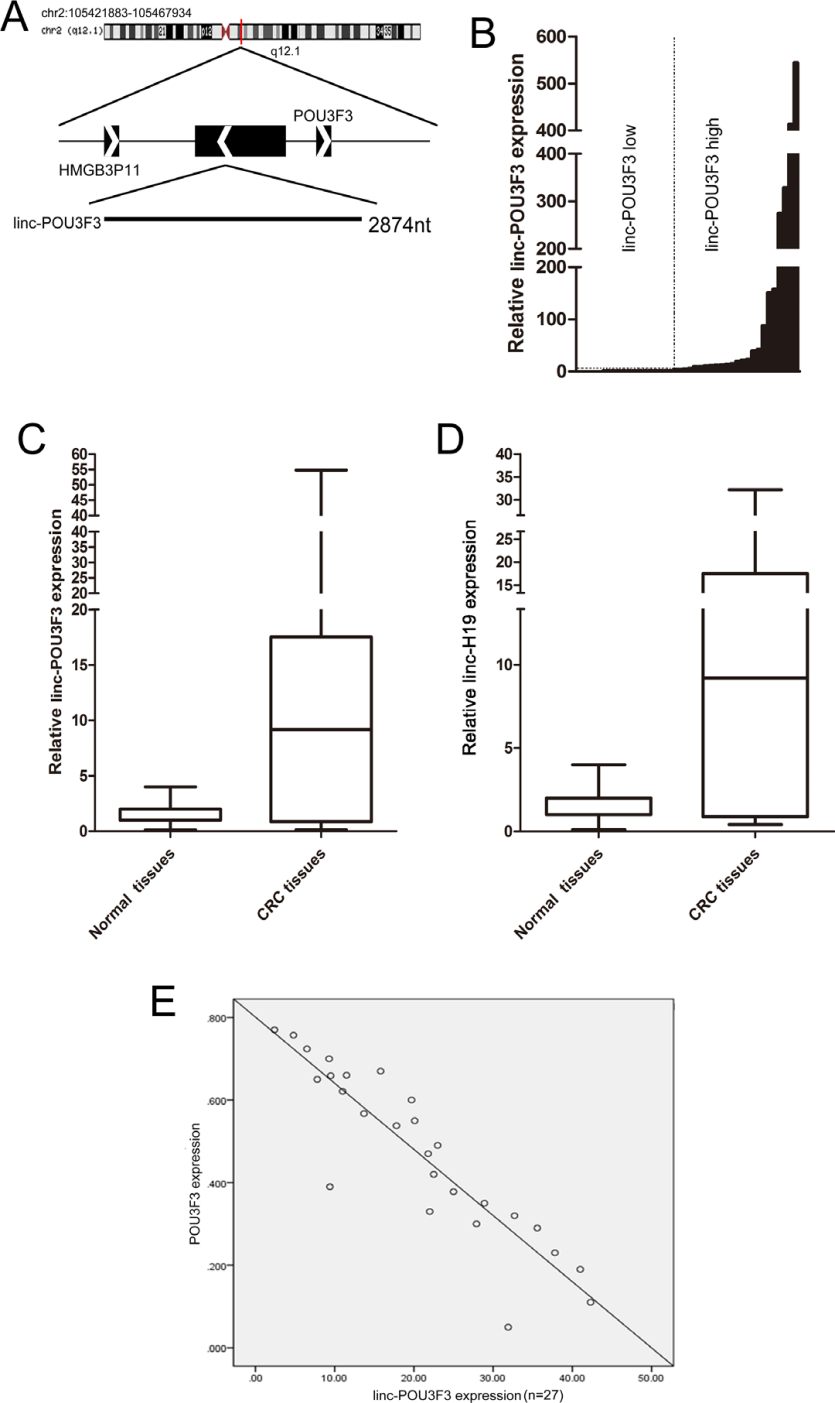
Abnormal linc-POU3F3 expression is associated with CRC **A.** Genomic location of linc-POU3F3 and its neighboring protein coding genes. **B.** QPCR analysis of the linc-POU3F3 expression level in 45 cases of CRC and adjacent non-tumor tissues. Twenty-eight cases (62.2%) showed increased expression, whereas 17 cases (37.8%) showed decreased expression, or no significant difference. **C.** and **D.** Twenty-eight cases of high linc-POU3F3 expression in 30 cases of linc-H19 high expression (fold change of ≥ 1.5; 93.0%), whereas 15 cases showing decreased or not significantly different linc-H19 expressions were observed among 17 cases of decreased or not significantly different linc-POU3F3 expression. Statistical difference was analyzed by the Wilcoxon signed-rank test (*P* < 0.01, Z = −3.684 for linc-POU3F3; *P* < 0.01, Z = −3.805 for linc-H19). A fold change of ≥ 1.5 was defined as overexpression (linc-POU3F3 high), and the rest was indicated as linc-POU3F3 low. **E.** The POU3F3 mRNA levels were plotted against linc-POU3F3 expression, and a significant inverse correlation was obtained (two-tailed Pearson's correlation, r = −0.894; *P* < 0.01).

Information from UCSC Browser showed that linc-POU3F3 is a transcript of 2,874 bp expressed from human chromosome 2 q12.1, comprising four exons on the reverse strand, and lying upstream from the POU3F3 gene, which is a member of the class III POU family of transcription factors (Fig. 1A). The expression level of linc-POU3F3 was assessed in 45 paired CRC samples and histologically normal adjacent tissues using quantitative real-time PCR (qPCR), with normalization to GAPDH. Compared with their normal counterparts, linc-POU3F3 expression was increased in cancerous tissues (fold change ≥ 1.5) in 28 cases (62.2%), whereas its expression was decreased or showed no significant difference in 17 cases (37.8%; Fig. 1B). Examination of the correlation between linc-POU3F3 expression and clinical pathological features showed that increased linc-POU3F3 expression correlated with the tumor histology grade and N grade (Table 1). However, linc-POU3F3 expression did not correlate with patients' gender, age, tumor size, T grade or M grade (Table 1).

**Table 1:** Association between patients, characteristics and Linc-POU3F3 expression in 45 CRC cases

Characteristics	Number of Patients *n* (%)	Linc-POU3F3		Chi-square	*p*-value
		Low	High		
Total	45	17 (37.8%)	28 (62.2%)		
Gender					
Male	22 (48.9%)	11	11	2.735	0.098
Female	23 (51.1%)	6	17		
Age (years)					
< 55	19 (42.2%)	9	10	2.003	0.175
≥ 55	26 (57.8%)	7	19		
Tumor size (cm)					
< 5	22 (48.9%)	6	16	2.021	0.155
≥ 5	23 (51.1%)	11	12		
Histology grade					
Well and moderate	33 (73.3%)	9	24	4.255	**0.039**
Poor	12 (26.7%)	8	4		
pT grade					
Ta, Tis, T1	4 (8.89%)	2	2	0.000	1.000
T2-T4	41 (91.1%)	15	26		
pN grade					
N0	22 (48.9%)	12	10	5.148	**0.023**
N1, N2	23 (51.1%)	5	18		
pM grade					
M0	25 (55.6%)	12	13	2.501	0.114
M1	20 (44.4%)	5	15		

Well and moderate: well and moderately differentiated; poor: poorly differentiated. Significant associations are shown in bold face in the *p*-value column.

Furthermore, linc-H19 was suggested to be tightly linked to tumorigenesis and to be prognostic significant for cancer progression in CRC [23, 24]; therefore, we compared the prognostic data of linc-H19 with that of linc-POU3F3 within these 45 cases CRC patients to assess the prognostic value of linc-POU3F3. The results showed that in 30 CRC tissues with high expression of linc-H19, 28 cases showed high expression of linc-POU3F3 (fold change of ≥ 1.5; 93.0%). On the other hand, in 17 CRC tissues with low expression of linc-POU3F3, 15 showed low expressions of linc-H19. The expressions of both linc-POU3F3 and linc-H19 were significantly elevated in the CRC tissues compared with the adjacent non-tumor tissues (*P* < 0.01, Z = −3.684 for linc-POU3F3; *P* < 0.01, Z = − 3.805 for linc-H19; Fig. 1C, 1D). Additionally, previous studies noted that the POU3F3 mRNA level was decreased in various cancers; therefore, we plotted the POU3F3 mRNA levels against linc-POU3F3 expression. We observed a significant inverse correlation between POU3F3 expression and the linc-POU3F3 level (two-tailed Pearson's correlation, r = −0.894; *P* < 0.01; Fig. 1E). This result implied that linc-POU3F3 overexpression might participate in the development of CRC and might serve as a novel marker for poor prognosis or progression of CRC.

### Knockdown of linc-POU3F3 levels in CRC cells

**Figure 2:**
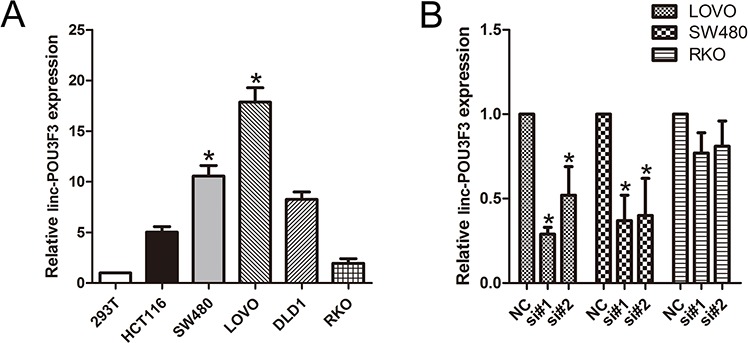
Knockdown of linc-POU3F3 levels in CRC cells **A.** QPCR analysis to examine the expression levels of linc-POU3F3 in various CRC cell lines (HCT-116, SW480, LOVO, DLD-1, and RKO) and in HEK293T cells (Mean ± SD, *n* = 3; *P < 0.05 *vs*. 293T). **B.** The knockdown efficiency in LOVO, SW480, and RKO cells by transfected si-linc-POU3F3 (NC, control siRNA; Mean ± SD, *n* = 3; *P < 0.05 *vs*. NC).

QPCR analysis was performed to examine the expression levels of linc-POU3F3 in various CRC cell lines (HCT-116, SW480, LOVO, DLD-1, and RKO) and in HEK293T cells (a human non-CRC cell line). LOVO and SW480 cells showed higher expression of linc-POU3F3; however, RKO showed lower expression of linc-POU3F3 (Fig. 2A). Thus, we used LOVO, SW480, and RKO cells as a model to investigate the effect of linc-POU3F3 on cell proliferation, apoptosis, migration and invasion.

We knocked down linc-POU3F3 expression in LOVO and SW480 cancer cells by transfection with siRNAs, si-linc-POU3F3. The knockdown efficiency in LOVO cells by si#1 and si#2 was 78.3% ± 7.4% and 66.3% ± 9.1%, respectively. In SW480 cells, the knockdown efficiency by si#1 and si#2 was 70.7% ± 10.2% and 60.7% ± 12.9%, respectively (Fig. 2B). However, the knockdown efficiency in RKO cells (low expression of linc-POU3F3) by si#1 and si#2 was 22.6% ± 3.4% and 19.5% ± 5.3%, respectively, which serve as a control.

### Linc-POU3F3 knockdown inhibited proliferation of CRC cells via cell cycle arrest

**Figure 3:**
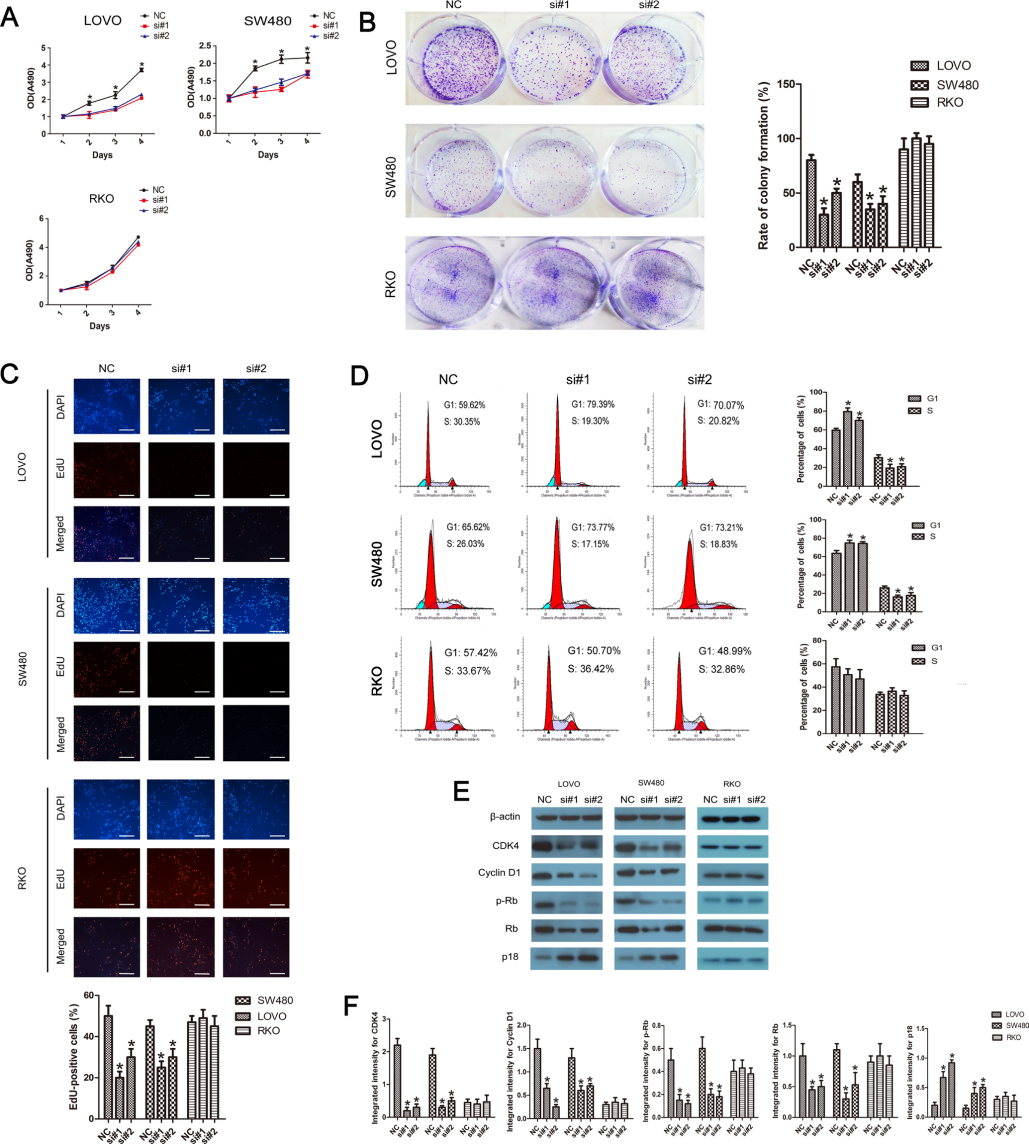
Linc-POU3F3 knockdown inhibited the proliferation of CRC cells via cell cycle arrest **A.** CellTiter 96 AQueous One Solution Cell Proliferation assay showing the proliferation in LOVO, SW480, and RKO cells after siRNA transfection. **B.** Histological analysis of the rate of colony formation in controls and linc-POU3F3 knockdown groups. **C.** The EdU incorporation assay to examine the effects of linc-POU3F3 inhibition on the DNA synthesis during cell growth. The images were taken at × 200. **D.** Flow cytometric analysis of cell cycle arrest 48 hours after treatment with siRNAs and negative controls in LOVO, SW480, and RKO cells. **E–F.** The expression of several important cell cycle-related proteins in linc-POU3F3 knockdown CRC cells. (Mean ± SD, *n* = 3; *P < 0.05 *vs*. NC).

As shown in Fig. 3A, siRNA-mediated knockdown of linc-POU3F3 impaired the proliferation in LOVO and SW480 cells, but not of in RKO cells, as revealed by the CellTiter 96 AQueous One Solution Cell Proliferation assay. The number of LOVO and SW480 cells was significantly decreased after transfection with si-linc-POU3F3 compared with the negative controls (*P* < 0.05). Consistent with these results, the ability to form colonies by LOVO and SW480 cells was also suppressed significantly after knockdown of linc-POU3F3 when compared with that by the negative controls (*P* < 0.05; Fig. 3B). RKO cells showed no difference in their colony forming ability after knockdown of linc-POU3F3 (*P* > 0.05; Fig. 3B). These results showed that linc-POU3F3 depletion had an obvious inhibitory effect on the growth of CRC cells.

A 5-ethynyl-2′-deoxyuridine (EdU) incorporation assay was used to examine the effects of linc-POU3F3 inhibition on DNA synthesis during cell growth. The proportion of S-phase cells (EdU positive cells) decreased in siRNA treated LOVO and SW480 groups compared with RKO group, suggesting that linc-POU3F3 depletion resulted in reduced DNA synthetic activity (*P* < 0.05; Fig. 3C). Furthermore, we transfected the cancer cells with siRNAs before analyzing the cell cycle distribution by flow cytometry. Both LOVO and SW480 cells treated with siRNAs showed apparent increases in the percentage of cells in the G1 phase, with concomitant decreases in the percentage of cells in the S phase, when compared with the negative controls (*P* < 0.05; Fig. 3D). RKO cells treated with siRNAs showed no difference compared with the control siRNA (*P* > 0.05; Fig. 3D), which was consistent with the EdU assay. These results proved that linc-POU3F3 knockdown led to cell cycle arrest in G1 phase, which might be responsible for the suppressed proliferation.

The knockdown of linc-POU3F3 led to an increased expression of p18 and a decreased expression of cyclin D1, cyclin-dependent kinase 4 (CDK4), phosphorylated retinoblastoma (Rb) and Rb in LOVO and SW480 cells (*P* < 0.05; Fig. 3E, 3F); The knockdown of linc-POU3F3 in RKO cells had no effect on these expressions compared with the control siRNA (*P* > 0.05; Fig. 3E, 3F). These results suggested that linc-POU3F3 promoted cell proliferation in CRC by regulating the cell cycle.

### Knockdown of linc-POU3F3 resulted in the intrinsic apoptosis in CRC cells

**Figure 4:**
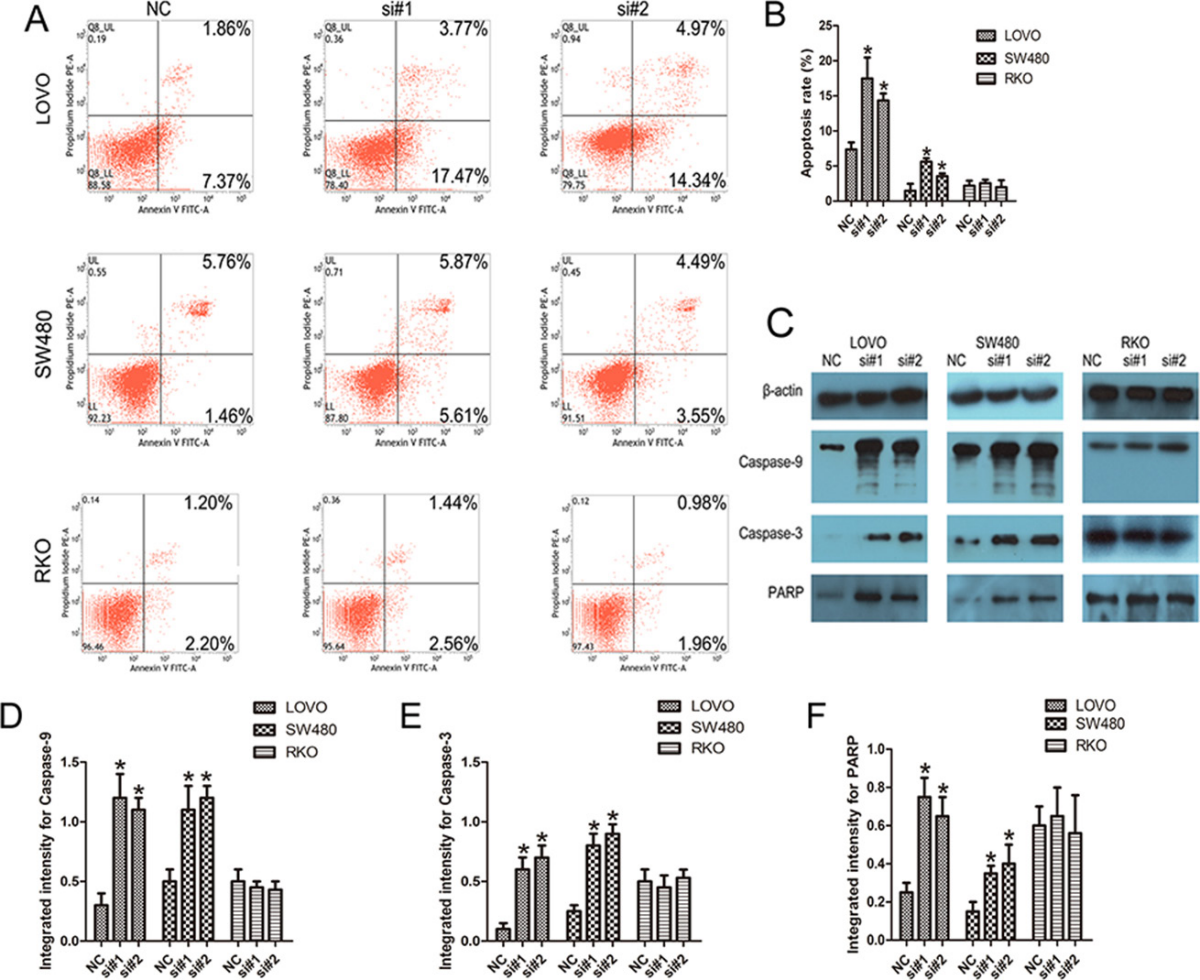
Knockdown of linc-POU3F3 resulted in the intrinsic apoptosis of CRC cells **A–B.** Flow cytometry analysis of the apoptotic rates in LOVO, SW480, and RKO cells after siRNAs treatment. **C–F.** Western blotting was used to investigate the alteration of apoptosis related proteins in LOVO, SW480, and RKO cells. (Mean ± SD, *n* = 3; *P < 0.05 *vs*. NC).

As shown by flow cytometry analysis in Fig. 4A and 4B, compared with the control cells, siRNAs treatment caused increased apoptosis in LOVO and SW480 cells, but not in RKO cells (*P* < 0.05). To explore the potential mechanisms accounting for the apoptosis-induced anticancer behaviors triggered by linc-POU3F3 depletion, Western blotting was conducted to investigate the expressions of apoptosis related proteins. Cleavages of caspase-9, caspase-7, and caspase-3 are prominent markers of the mitochondria-mediated, caspase-dependent pathway. In the present study, the increased rate of apoptosis after linc-POU3F3 knockdown was consistent with increased abundances of cleaved caspase-9, caspase-3, and poly (ADP-ribose) polymerase (PARP) in both LOVO and SW480 cells (*P* < 0.05; Fig. 4C–4F). However, the expressions of these apoptosis related proteins showed no difference after linc-POU3F3 knockdown in RKO cells (*P* > 0.05; Fig. 4C–4F). These results indicated that activation of the intrinsic apoptotic pathway was involved in the apoptosis induced by linc-POU3F3 knockdown.

### Knockdown of linc-POU3F3 inhibited migration and invasion in CRC cells

**Figure 5:**
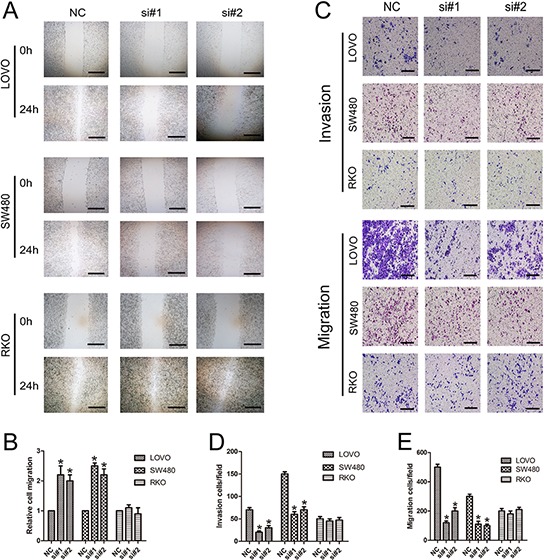
Knockdown of linc-POU3F3 inhibited migration and invasion in CRC cells **A.** Images of a wound healing assay after linc-POU3F3 knockdown in LOVO, SW480, and RKO cells at 24 h after scratching. **B.** The relative cell migrations of LOVO, SW480, and RKO cells at 24 h after scratching were showed in this panel. **C.** Transwell analysis migration and invasion abilities of CRC cells. **D–E.** Histological analysis of OD (570 nm) absorbance of crystal violet-stained cells in transwell assay. (Mean ± SD, *n* = 3; *P < 0.05 *vs*. NC).

We first explored the role of linc-POU3F3 knockdown in CRC cells migration via the wound healing assay. The si-linc-POU3F3-treated LOVO and SW480 cells showed comparatively slower migration towards the wound space; however, cells treated with control siRNA migrated more aggressively and nearly closed the wound at 24 h after scratching (Fig. 5A, 5B). The RKO cells showed the same migration activity as cells treated with si-linc-POU3F3 or control siRNA (Fig. 5A, 5B). The involvement of linc-POU3F3 in migration and invasion of CRC cells was also investigated by inhibiting linc-POU3F3 expression with siRNAs and then subjecting them to the transwell assay. Fig. 5C–5E shows that knockdown of linc-POU3F3 significantly inhibited migration and invasion abilities of LOVO and SW480 cells in the transwell assay (*P* < 0.05). By contrast, RKO cells showed no difference in their migration and invasion abilities after linc-POU3F3 knockdown (*P* > 0.05).

### Knockdown of linc-POU3F3 inhibited epithelial-mesenchymal transition (EMT) in CRC cells

**Figure 6:**
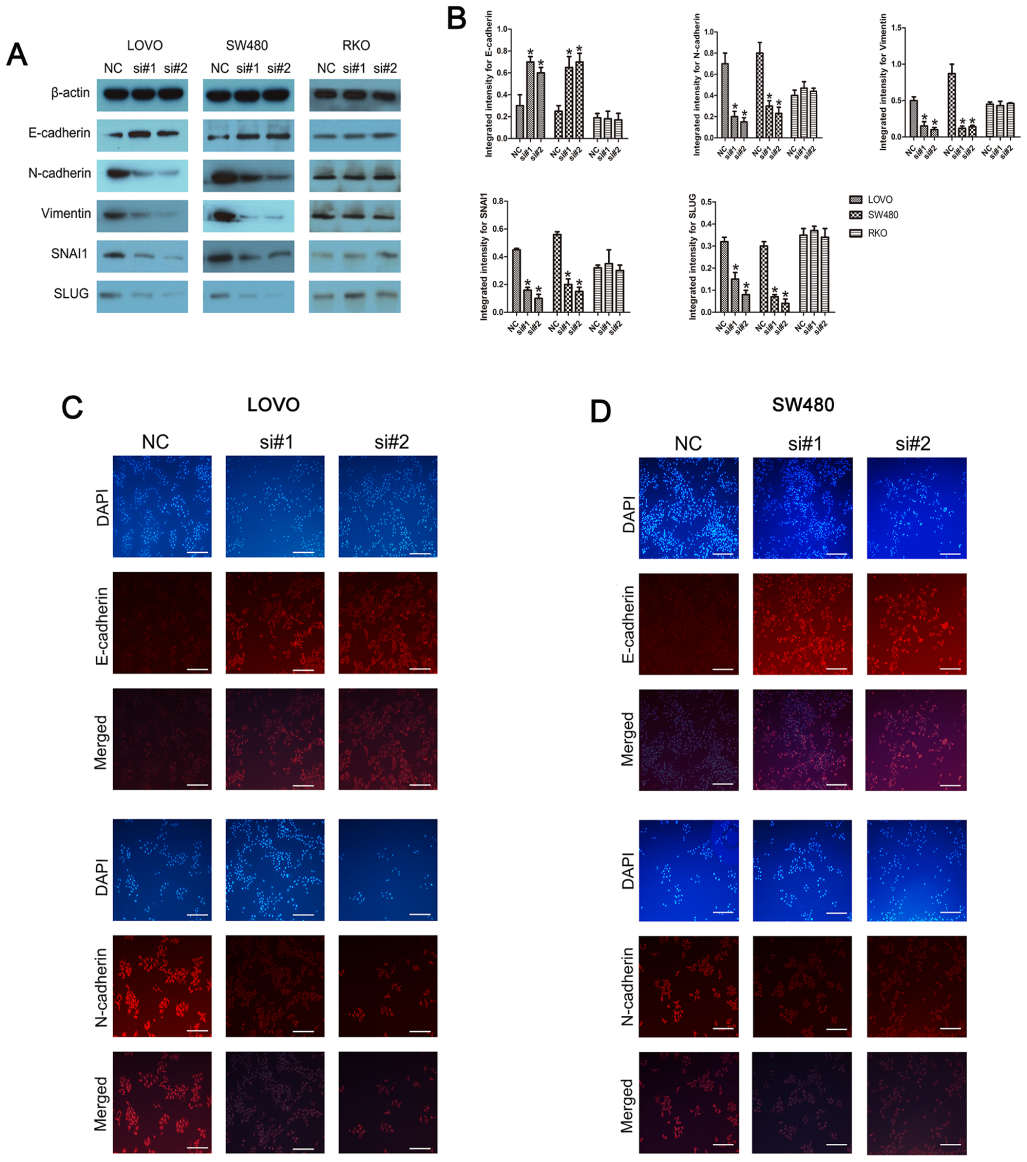
Knockdown of linc-POU3F3 inhibited EMT in CRC cells **A–B.** Western blotting was used to investigate the alteration in expression of epithelial and mesenchymal markers (E-cadherin, N-cadherin, Vimentin, SNAI1, and SLUG). **C–D.** Immunofluorescence images of CRC cells stained for E-cadherin and N-cadherin. The images were taken at × 200. DAPI, E-cadherin, and N-cadherin staining are shown separately and then the merged images are shown (Mean ± SD, *n* = 3; *P < 0.05 *vs*. NC).

Western blotting was used to investigate the alteration in the abundance of epithelial and mesenchymal markers. Linc-POU3F3 knockdown increased the abundance of the epithelial marker, E-cadherin, in LOVO and SW480 cells, while the amounts of mesenchymal markers, namely, N-cadherin and vimentin, were significantly reduced (*P* < 0.05; Fig. 6A, 6B). In addition, the amounts of SNAI1 and SLUG, two predominant regulators of the EMT process, were decreased upon knockdown of linc-POU3F3 in LOVO and SW480 cells (*P* < 0.05; Fig. 6A, 6B). The RKO cells showed no difference in expression of these EMT markers after linc-POU3F3 knockdown (*P* > 0.05; Fig. 6A, 6B). Furthermore, we performed an immunofluorescence assay to detect the expression of E-cadherin and N-cadherin. In LOVO and SW480 cells, linc-POU3F3 knockdown increased the expression of E-cadherin, but decreased the expression of N-cadherin (*P* < 0.05; Fig. 6B, 6C). These results indicated that linc-POU3F3 might contribute to EMT progression in LOVO and SW480 cells, thus aiding migration and invasion.

### Linc-POU3F3 knockdown induced activation of BMP and autophagy signaling

**Figure 7:**
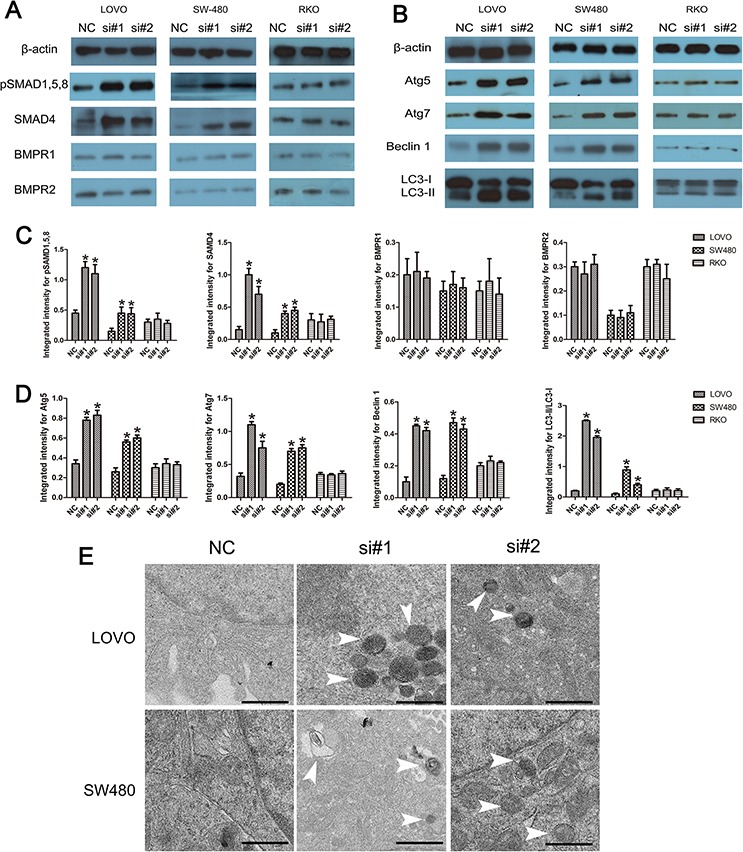
The involvement of BMP and autophagy pathway induced by linc-POU3F3 knockdown **A–B.** The protein expressions of BMP pathway (BMPR1, BMPR2, SMAD4, and pSMAD1, 5, 8) and autophagy pathway (Atg5, Atg7, Beclin 1, and LC3) induced by linc-POU3F3 knockdown in LOVO, SW480, and RKO cells were determined by Western blotting. **C–D.** The protein levels of BMP pathway and autophagy pathway in LOVO, SW480, and RKO cells were showed in these panels. - **E.** TEM showing the formation of autophagosomes after siRNA treatment in LOVO and SW480 cells. Representative images of autophagosomes are shown at the bottom (white arrowheads). The images were taken at × 5000. (Mean ± SD, *n* = 3; *P < 0.05 *vs*. NC).

As revealed by Western blotting, knockdown of linc-POU3F3 increased the abundance of pSMAD1, 5, 8 and SAMD4 in LOVO and SW480 cells (*P* < 0.05; Fig. 7A, 7C). In contrast, the amounts of BMPR1 and BMPR2 were not significant different after treatment with si-linc-POU3F3 (*P* > 0.05; Fig. 7A, 7C). The RKO cells showed no difference in their amounts of BMPRs, SMAD4, and pSMAD1, 5, 8, after linc-POU3F3 knockdown (*P* > 0.05; Fig. 7A, 7C). These results demonstrated that linc-POU3F3 knockdown increased the abundance of pSMAD1, 5, 8 and SAMD4 to activate the BMP signaling.

Knockdown of SMAD4 inhibited autophagy, and cell autophagy occurred earlier than execution of apoptosis [18]. In this study, in LOVO and SW480 cells, Western blotting demonstrated the accumulation of Atg5, Atg7, and Beclin 1 in LOVO and SW480 cells after siRNA treatment (*P* < 0.05; Fig. 7B, 7D). In addition, the amount of LC3-II increased after transfection with siRNA. However, the LC3-I level was decreased compared with the negative control siRNA (*P* < 0.05; Fig. 7B, 7D). The RKO cells showed no difference in their expressions of autophagy-related proteins after linc-POU3F3 knockdown (*P* > 0.05; Fig. 7B, 7D). In addition, transmission electron microscopy (TEM) showed that the formation of autophagosomes increased after linc-POU3F3 knockdown in LOVO and SW480 cells, which were recognized as characteristic double-membrane vacuolar structures containing various kinds of cytoplasmic contents (Fig. 7E).

## DISCUSSION

The treatment for human cancer remains a huge challenge because of the unlimited proliferation, defective apoptosis and metastasis of cancer cells. In recent years, studies have revealed that dysregulation of lincRNAs might affect epigenetic information and provide a cellular growth advantage [25]. However, for most of these lincRNAs, the detailed functions, mechanisms, and signaling pathways through which they exert their biological functions are not well understood. Therefore, we conducted the present study to clarify the possible relationships between CRC and linc-POU3F3 and to explore the potential application of linc-POU3F3 in the diagnosis and treatment of CRC.

We demonstrated that linc-POU3F3 was overexpressed in CRC tissues compared with the adjacent non-tumor tissues and positively correlated with the tumor histology grade and N grade. Linc-H19 was the widely investigated in many CRC studies, and has good prognostic significance in CRC [23, 24]. Thus, we compared the prognostic significance for CRC between linc-H19 and linc-POU3F3 and found that linc-POU3F3 was comparable with linc-H19. Previous studies demonstrated that increased levels of linc-POU3F3 promote POU3F3 DNA methylation, leading to reduced POU3F3 mRNA levels in ESCC [21]. In this study, we observed that low expression of POU3F3 was inversely correlated with linc-POU3F3 levels in CRC specimens (Fig. 1). Taken together, the results suggested that linc-POU3F3 is a useful diagnostic biomarker or therapeutic target in CRC [26]. However, the association between linc-POU3F3 expression levels and the overall survival of patients remains unclear, which might reflect the limited number of cases and follow-up time. Prospective studies in larger cohorts are needed.

The role of linc-POU3F3 in CRC was further investigated by detecting the alterations of biological behaviors in CRC cell lines after linc-POU3F3 knockdown. Knockdown of linc-POU3F3 resulted in suppressed proliferation in LOVO and SW480 cells, concomitant with induction of cell cycle arrest, apoptosis and inability to metastasize (Fig. 3, 4, 5). Knockdown of linc-POU3F3 in RKO cells, which have low expression of linc-POU3F3, caused no significant differences in proliferation, apoptosis, and metastatic ability, which further validated the role of linc-POU3F3 in the biological behavior of CRC cell lines.

Cancer progression is commonly associated with disorders in cell cycle control, which leads to the unlimited proliferation of cancer cells [27, 28]. The cell cycle transition from the G1 phase to the S phase is the major regulatory checkpoint in cell proliferation. In this study, flow cytometry analysis and EdU incorporation assays showed that linc-POU3F3 knockdown induced cell cycle arrest at the G1 phase and lowered the percentage of LOVO and SW480 cells in the S phase (Fig. 3). We then evaluated the expressions of proteins that were correlated with G1 phase and the G1/S transition of the cell cycle to explore the mechanism underling the observed proliferation alterations after linc-POU3F3 knockdown. Knockdown of linc-POU3F3 inhibited the expressions of cyclin D1, CDK4, and p-Rb, accompanied by a decrease in total Rb, and increased the expression of p18 (Fig. 3). Cyclin D1 promote cells to enter the G1 phase by activating CDK4, which leads to increased phosphorylation of Rb (p-Rb) [29, 30]. The Ink4 (Inhibitor of CDK4) family, such as p15 (INK4B) and p18 (INK4C), can specifically inhibit the complex of CDK4-cyclin D to decrease the phosphorylation of Rb to regulate cell cycle [31]. Thus, the mechanism underlying the growth arrest might involve increased p18 expression, which lead to an inhibition of the complex of CDK4-cyclin D1 and phosphorylation of Rb, and ultimately induced cell cycle arrest at the G1 phase. The cell cycle arrest was attributed, at least in part, to the anticancer effect of linc-POU3F3 knockdown on tumor growth. Collectively, the above results revealed the vital role of linc-POU3F3 in promoting tumorigenesis and progression of CRC. Linc-POU3F3 could be a potential therapeutic target in CRC.

Defective apoptosis is one of the hallmarks of cancer cells. In the process of cell apoptosis, the caspase family is indispensable for the initiation and execution of cell death in response to various kinds of stimuli [32–34]. The upregulation of intrinsic apoptotic signal recruits and activates initiator caspase-9 and effector caspases (caspase3/6/7), ultimately resulting in cellular death. Knockdown of linc-POU3F3 by siRNA induced apoptosis of CRC cells by activating caspase-9 and caspase-3 (Fig. 4), indicating that linc-POU3F3 inhibition might enhance the chemosensitivity of CRC cells.

Metastasis of cancer is the major cause of death among cancer patients [35–37]. In our study, wound healing and transwell analyses demonstrated that knockdown of linc-POU3F3 expression markedly weakened the migration and invasion ability of LOVO and SW480 cells compared with the negative control (Fig. 5). Aberrant activation of the EMT program contributes to the initiation of the multistep metastatic process. Downregulation of the epithelial marker E-cadherin induced the expressions of certain mesenchymal markers, such as N-cadherin and Vimentin, during EMT [38]. Our study revealed that after linc-POU3F3 knockdown, the protein expressions of mesenchymal markers were significantly decreased, while epithelial markers significantly increased compared with the negative controls in LOVO and SW480 cells (Fig. 6). These results indicated that linc-POU3F3 might promote EMT progression in CRC cells.

Various factors may influence metastatic ability of cancer cells through different signaling pathways [39, 40]. SMAD4, as a major factor of the BMP pathway, participates in variety physiological and pathological processes, including metastasis [41, 42]. In this study, we showed that inhibition of linc-POU3F3 resulted in overexpression of SMAD4 and pSMAD1, 5, 8, in LOVO and SW480 cells (Fig. 7). Based on above results, increased BMP signaling after inhibition of linc-POU3F3 resulted in reduced migration and invasion capacities of CRC cells.

In addition to the BMP pathway and cancer metastasis, we revealed a novel regulatory function of linc-POU3F3 in autophagy within CRC cells. Although autophagy might allow tumor cells to survive under metabolic stress, associations between defects of autophagy and the development of cancer have been suggested genetically [43]. Moreover, autophagy and apoptosis might be linked to each other and occur simultaneously or sequentially in a cell type-, death stimulus-, and context-dependent manner [44–46]. SMAD4 has an important function in autophagy signaling and SMAD4 knockdown abolished TGF-β-induced activation of autophagy-related proteins [47, 48]. We showed, for the first time, that linc-POU3F3 knockdown resulted in an increased level of SMAD4, which might partly contribute to the higher autophagy activities. Consistent with this, we found that linc-POU3F3 knockdown activated autophagy more potently and continuously by significantly increasing the expressions of BECLIN1, ATG5, ATG7, and LC3 II in LOVO and SW480 CRC cells compared with the negative control (Fig. 7). These results indicated a new regulatory function of linc-POU3F3 in autophagy. Although our findings revealed the involvement of SMAD4 in autophagy after linc-POU3F3 inhibition, further investigations are required to test this hypothesis.

In conclusion, our study showed that in LOVO and SW480 cells, linc-POU3F3 promoted proliferation and metastasis, while attenuating apoptosis. The potential mechanisms underlying these effects included promoting transition of the cell cycle, inactivation of intrinsic apoptosis, and aiding metastasis, which involved in BMP and autophagy signaling pathways. In addition, positive correlation between linc-POU3F3 expression and clinical parameters, including tumor grading and N stage were detected. Thus, linc-POU3F3 could be a promising therapeutic target and novel molecular biomarker for CRC.

## MATERIALS AND METHODS

### Patients and tissue samples of CRC

Forty-five paired samples of CRC and adjacent non-tumor tissues (more than 5 cm away from the tumor) were obtained from patients who received surgery in the Sun Yat-Sen Memorial Hospital of Sun Yat-Sen University. The postoperative pathological staging of each subject was determined according to the 7th edition of the Union for International Cancer Control (UICC) tumor-node-metastasis (TNM) staging system for CRC. Tissue samples were collected, immediately snap frozen in liquid nitrogen and stored at −80°C until further analysis. Before the use of these clinical materials for research purposes, written consents from all patients and approval of the Hospital Ethic Review Committees were obtained.

### Cell lines and culture conditions

Five CRC cell lines (DLD1, HCT116, LOVO, RKO, and SW480) and the 293T cell line were purchased from the Institute of Biochemistry and Cell Biology of the Chinese Academy of Sciences (Shanghai, China). Cells were cultured in RPMI 1640 or DMEM (Gibco, Grand Island, NY, USA) medium supplemented with 10% fetal bovine serum (10% FBS), 100 U/ml penicillin, and 100 mg/ml streptomycin (Gibco) in humidified air at 37°C with 5% CO2.

### RNA extraction and real-time quantitative polymerase chain reaction (QPCR)

Total RNA from cell lines and tissue samples was extracted using the Trizol reagent (Invitrogen). First-strand cDNA was synthesized with PrimeScript RT Master Mix (TAKARA, Dalian, China). After reverse transcription of the total RNA, qPCR was conducted to examine the expression of linc-POU3F3, using SYBR Green PCR Master mix (TAKARA) on a Bio-Rad Real-Time PCR instrument (Bio-Rad, Hercules, California, USA). GAPDH was used as an internal reference gene to normalize RNA levels between different samples for an exact comparison of transcription levels. The sequences of primers were as follows (in the 5′ to 3′ orientation): GAPDH forward, GGG AGC CAA AAG GGT CAT, GAPDH reverse, GAG TCC TTC CAC GAT ACC AA; linc-POU3F3 forward, AAT CAC TGC AAT TGA AGG AAA AA, linc-POU3F3 reverse, CCT TGT TTT CCA ACC CTT AGA CT; linc-H19 forward, TGC TGC ACT TTA CCA A CTG, linc-H19 reverse, ATG GTG TCT TTG ATG ATG TTG GCC. Data were analyzed using the ΔΔCt (cycle threshold) method with GAPDH as the constitutive marker [49].

### Knockdown of linc-POU3F3 expression

In the transient transfection experiments, small interfering RNAs (siRNAs) targeting linc-POU3F3 (si#1: GCU GUU CAA UAC AGC UCU UTT, AAG AGC UGU AUU GAA CAG CTT. si#2: CCG GUA UAC CAU AAU GCU UTT, AAG CAU UAU GGU AUA CCG GTT) and the negative control siRNA (UUC UCC GAA CGU GUC ACG UTT, ACG UGA CAC GUU CGG AGA ATT) were used (GenePharma, Shanghai, China). RKO, LOVO and SW480 cells were seeded at a density of 1.5 × 10^5^ cells per well in 6-well plates. Twenty-four hours later, cells were transfected with siRNAs using Lipofectamine 3000 Transfection Reagent (Life Technologies, Grand Island, NY, USA), following the manufacturer's instructions. The effectiveness of siRNA knockdown was assessed by qPCR.

### EdU incorporation assay

To assess cell proliferation, cells were seeded in 24-well plates. The cells were incubated under standard conditions in complete media. Forty-eight hours after transfection, cell proliferation was detected using an EdU Cell Proliferation Assay Kit (Ribobio, Guangzhou, China). Briefly, the cells were incubated with 50 mM EdU for 3 h before fixation, permeabilization, and EdU staining, which were performed according to the manufacturer's protocol. The cell nuclei were stained with DAPI (Sigma-Aldrich, St. Louis, MO, USA) at 1 mg/mL for 10 min. The proportion of cells that incorporated EdU was determined using fluorescence microscopy.

### CellTiter 96 Aqueous One Solution Cell Proliferation assay

The effect of linc-POU3F3 knockdown on cell growth in RKO, LOVO, and SW480 cell lines were measured using a CellTiter 96 Aqueous One Solution Cell Proliferation Assay kit (Promega, Madison, WI, USA). Cells were collected at 24 h after treatment of siRNAs. 1000 cells per well were seeded in 96-well plates and allowed to adhere overnight. On the second day, CellTiter 96 Aqueous One Solution was added to at least five replicate wells at one-fifth of the total volume and the cells were incubated for 2 hours at 37°C. Absorbance was measured using a multifunctional microplate reader SpectraMax M5 (Molecular Devise, Sunnyvale, CA, USA) at 490 nm. The measurement of cell proliferation was conducted every 24 hours, and lasted 4 days. Cell growth curves were constructed with absorbance as the ordinate and time as the abscissa.

### Colony formation assay

RKO, LOVO, and SW480 cells, treated by siRNAs and negative control for 24 hours, were routinely trypsinized and seeded in 6-well plates (1000 cells/well). The medium was changed every three days. After one week, the cells were washed with PBS, fixed with 4% paraformaldehyde for 30 minutes, and then stained with crystal violet for 30 minutes for visualization and counting.

### Flow cytometry assay

Flow cytometry analysis was performed to determine whether suppression of linc-POU3F3 could inhibit the growth phase of CRC cells. RKO, LOVO, and SW480 cells were seeded into 6-well plates. Forty-eight hours after transfection, the cells were harvested and stained with annexin V-FITC and propidium iodide (PI), according to the manufacturer's instructions. The cellular apoptotic rate was evaluated using a FACS VerseTM flow cytometer (Becton Dickinson, CA, USA). Cells for growth phase analysis were resuspended in 200 μl PBS, fixed with 70% ice-cold ethanol overnight, and stained with PI. The cell cycle was detected by the FACSVerse™ flow cytometer.

### Wound healing assay

A wound healing assay was used to test the migration ability of CRC cells. In our study, 2 × 10^5^ cells were plated in 24-well plates after transfection by linc-POU3F3 and siRNA control for 48 h. When cell confluence reached approximately 100%, the old medium was removed and the monolayer was wounded by scratching with a 10-μl sterile pipette tip lengthwise along the chamber. The cells were then washed three times with PBS and cultured with serum-free medium at 37°C. Images of cells migrating into the wound were photographed at 0 h, 24 h, 48 h, and 72 h using an inverted microscope. Wound width (μm) was measured using Image J software.

### Migration and invasion assay

Cell migration and invasion capacity were measured using transwell migration assays (Millipore, Billerica, MA) *in vitro*. RKO, LOVO, and SW480 cells were transfected with linc-POU3F3 and siRNA control for 48 h, and then suspended in RPMI-1640 at a density of 1 × 10^6^ cells / mL. The cell suspensions (150 μL) were then seeded in the upper chamber with a porous membrane coated with (for the transwell invasion assay) or without (for the migration assay) Matrigel (BD Bioscience, San Diego, CA). To attract the cells, 500 μL of RPMI-1640 with 10% serum was added to the bottom chamber. After allowing the cells to migrate for 24 h or to invade for 48 h, the penetrated cells on the filters were fixed in dried methanol and stained in 4 g/L crystal violet. The numbers of migrated or invasive cells were determined from five random fields using a microscope (Nikon, Tokyo, Japan).

### Transmission electron microscopy (TEM)

Specimens were immersed in 2% cacodylate-buffered glutaraldehyde for 6 h. They were then rinsed in cacodylate buffer supplemented with 15% sucrose, post fixed with 1% phosphate-buffered OsO4 (pH 7.4) for 2 h, dehydrated with alcohol, clarified in propylene oxide, and embedded in Epon. Ultrathin sections were made using an ultramicrotome, and stained with uranyl acetate, followed by a saturated solution of bismuth subnitrate and finally examined under a JEM 1400 electron microscope (Hitachi, Tokyo, Japan).

### Immunofluorescence

Cells (1.0 × 10^4^ cells/well) were seeded into 24-well culture plates, followed by transfection with siRNAs to knockdown linc-POU3F3 expression. Forty-eight hours after transfection. The cells were incubated with mouse anti-E-cadherin and anti- N-cadherin (1:100; Cell Signaling Technology, Beverly, MA, USA) antibodies at 4°C overnight followed by washing with PBS three times. Coverslips were then incubated with Texas Red-conjugated anti-rabbit antibodies (1:200; Life Technologies, Grand Island, NY, USA) for 30 min at room temperature, and then stained with DAPI (1:200; Promega).

### Protein extraction and western blotting

Cells were rinsed twice with cold PBS and lysed by RIPA buffer (Thermo Fisher Scientific, Waltham, MA, USA) containing protease inhibitor cocktail (Roche). Protein (40 μg per sample) was separated by SDS-PAGE using a 10% polyacrylamide gel. The proteins were transferred electrophoretically onto a PVDF membrane. Blotted membranes were blocked in 5% skimmed milk diluted in TBST, followed by incubation with appropriate primary antibodies (anti-cyclin D1, CDK4, p18, Rb, p-Rb, caspase-9, caspase-3, PARP, E-cadherin, N-cadherin, Vimentin, SNAI1, SLUG, BMPR1, BMPR2, SMAD4, pSMAD1, 5, 8, Atg5, Atg7, Beclin 1, LC3, and β-actin; obtained from Cell Signaling Technology and all the antibodies were diluted 1:1000.) overnight at 4°C. The membranes were then washed for 5 minutes for three times with TBST, and subsequently incubated for 1 hour with HRP-linked secondary antibody (Cell Signaling Technology) at room temperature. β-actin was used as an internal control. The blots were detected using an enhanced chemiluminescence kit (Millipore) and subjected to autoradiography using X-ray film.

### Statistical analysis

All the experiments were performed at least three times, and then mean values and standard deviation (SD) were calculated. Differences between two groups were analyzed by Student's *t*-test. The correlation between linc-POU3F3 expression and the clinical characteristics of the CRC samples was determined using Pearson's Chi-square test in SPSS 22.0. A value of *P* < 0.05 was considered to be statistically significant.
